# Sleep increases chromosome dynamics to enable reduction of accumulating DNA damage in single neurons

**DOI:** 10.1038/s41467-019-08806-w

**Published:** 2019-03-05

**Authors:** D. Zada, I. Bronshtein, T. Lerer-Goldshtein, Y. Garini, L. Appelbaum

**Affiliations:** 1 The Faculty of Life Sciences and the Multidisciplinary Brain Research Center, Bar-Ilan University, Ramat-Gan, 5290002 Israel; 2 Department of Physics and the Institute for Nanotechnology, Bar-Ilan University, Ramat-Gan, 5290002 Israel

## Abstract

Sleep is essential to all animals with a nervous system. Nevertheless, the core cellular function of sleep is unknown, and there is no conserved molecular marker to define sleep across phylogeny. Time-lapse imaging of chromosomal markers in single cells of live zebrafish revealed that sleep increases chromosome dynamics in individual neurons but not in two other cell types. Manipulation of sleep, chromosome dynamics, neuronal activity, and DNA double-strand breaks (DSBs) showed that chromosome dynamics are low and the number of DSBs accumulates during wakefulness. In turn, sleep increases chromosome dynamics, which are necessary to reduce the amount of DSBs. These results establish chromosome dynamics as a potential marker to define single sleeping cells, and propose that the restorative function of sleep is nuclear maintenance.

## Abstract (web summary)

Do single neurons require sleep and what is the conserved cellular function of sleep? In this paper, the authors use real-time imaging of chromosomes in individual cells within live zebrafish to show that sleep increases chromosome dynamics, which are necessary to reduce DNA damage that is accumulated during wakefulness.

## Introduction

Sleep is vital to animal life and is found in all studied animals, ranging from jellyfish to worm, fly, zebrafish, rodents, and humans^1–5^. Prolonged sleep deprivation can be lethal, and sleep disturbances are associated with various deficiencies in brain performance^6^. Sleep is regulated by circadian and homeostatic processes^7^, and is coupled with reduced awareness of the environment and a high risk for survival. Several mechanisms can explain the roles of sleep, ranging from macromolecule biosynthesis, energy conservation, and metabolite clearance, to synaptic plasticity and memory consolidation^8–12^. However, why sleep has evolved and which fundamental ancestral functions it regulates, remain enigmatic.

In mammals and birds, sleep is defined by behavioral criteria and cycles of electroencephalographic (EEG) patterns, which differentiate between wakefulness and sleep states. In non-mammalian animals, including zebrafish, sleep is solely defined by behavioral criteria, such as periods of immobility associated with a species-specific posture and an increased threshold of arousal to external stimuli^13–16^. In all animals, including animals with simple neuronal networks^2,3^, circuits of sleep- and wake-promoting neurons orchestrate the behavioral states^17^. Evidence across multiple animals supports the notion that sleep can occur locally in the brain^18^ or perhaps even in a small number of cells^19^. Nevertheless, although sleep significantly contributes to the overall temporal organization of the transcriptome^20,21^, there are no molecular markers that can be reliably used across phylogeny to define sleep in a single cell^22^.

The nuclear architecture and the dynamic changes in chromatin organization regulate vital cellular processes, including epigenetics, genomic stability, transcription, cell cycle, and DNA replication and repair^23,24^. Chromatin dynamics, such as chromosome movements and structural genomic arrangements, are regulated by proteins that interact with the nuclear lamina and envelope in dividing cells^25–27^. In mature and non-dividing neurons, the role of chromatin dynamics is less understood^28^. Accumulating evidence showed that chromatin remodeling is implicated in circadian function. The changes in chromatin organization and epigenetic landscape shape the expression profile of a large number of rhythmic genes^29^. However, the effect of sleep on chromatin dynamics in neurons is unknown.

Recent works showed that sleep can be induced by cellular stress in *Caenorhabditis elegans* and mammals^30,31^. Moreover, sleep has been associated with the faster repair of DNA double-strand breaks (DSBs) in mice and fruit flies^32^. The causes of DSBs are diverse and include reactive oxygen species (ROS), ionizing radiation, and inadvertent action of nuclear enzymes^33^. Notably, neuronal activity can also induce DSBs. In specific mouse neurons, DSBs can be generated by physiological brain activity during natural exploration of the environment^34^. Furthermore, activity-induced DSBs facilitate the expression of immediate early genes in mouse and cell cultures, possibly because they resolve topological constraints in the genome^35^.

We hypothesized that sleep has evolved in order to enable single neurons to perform nuclear maintenance. To test which nuclear process favors sleep time, real-time imaging of chromosome dynamics, neuronal activity as well as quantification of DSBs and sleep, were coupled with genetic and pharmacological manipulations in live zebrafish. The findings propose a definition for a single sleeping neuron; i.e., increased chromosome dynamics, and suggest a role for sleep; i.e., nuclear maintenance.

## Results

### Imaging chromosome dynamics in live larvae

**Fig. 1:**
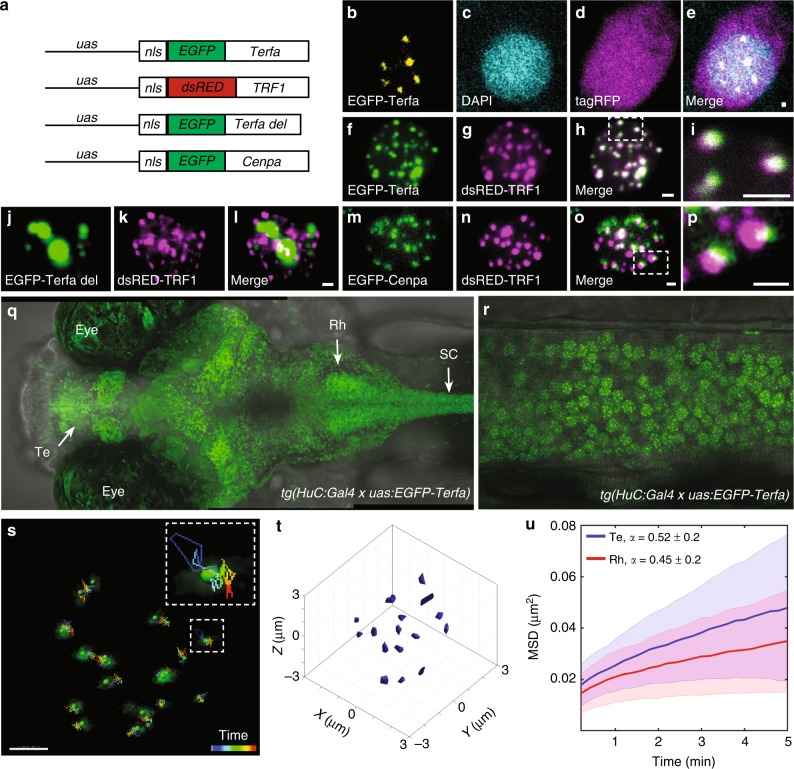
Imaging single chromosome dynamics in live larvae. **a** The DNA constructs were used to express telomere, centromere, and truncated telomere markers. **b**–**e** One-plane view of a representative neuron that expresses cellular tagRFP (magenta) and nuclear EGFP-Terfa (yellow) telomeric markers in live larvae. **f**–**i** Co-localization of zebrafish (**f**) and human (**g**) telomeric markers in the nucleus of a zebrafish neuron. **j**–**l** Co-expression of truncated zebrafish (**j**) and human (**k**) telomeric markers. **m**–**p** Co-expression of centromere (**m**) and telomere (**n**) markers. Dorsal (**q**) and lateral (spinal cord, **r**) views of 6-dpf *tg(HuC:Gal4)*/*tg(uas:EGFP-Terfa)* larvae (Te telencephalon, Rh rhombencephalon, SC spinal cord). **s** Live time-lapse imaging of a single neuron nucleus shows the movement of chromosomes measured over 9.5 min. Dashed box shows high magnification of a single trajectory. **t** 3D single-particle tracking (SPT) shows the volume of motion of chromosomes in a single nucleus. **u** In all Te and Rh neurons, the time-averaged mean square displacement (MSD) for telomeres trajectory (Te: *n* = 340 chromosomes, Rh: *n* = 475 chromosomes). Lines represent the means, and the shaded area represents the standard deviation (SD). Scale bar = 1 µm

Chromatin dynamics constitute a fundamental component of genome regulation and cell function^36^. In order to visualize and quantify chromosome dynamics in live zebrafish, the zebrafish *telomeric repeat binding factor a* (*terfa*) was cloned, and the telomere marker EGFP-Terfa was expressed in zebrafish neurons, resulting in a nucleus-specific punctum pattern (Fig. 1a–e). To verify that EGFP-Terfa marks chromosomes, the human telomere marker *uas:dsRED-TRF1*^37^ was co-injected with either *uas:EGFP-Terfa* or the DNA binding-site-deleted construct *uas:EGFP-Terfa del* into one-cell-stage *tg(HuC:Gal4)* embryos. While zebrafish and human telomeric markers co-localized (Fig. 1f–i), deletion of the Terfa DNA binding site resulted in non-specific protein aggregates in the nucleus (Fig. 1j–l). To further validate that the puncta mark chromosomes, the zebrafish *centromere protein a (cenpa*) was cloned, and the EGFP-Cenpa was used as a centromere marker. Two-color imaging showed that EGFP-Cenpa and dsRED-TRF1 puncta were expressed adjacently on the chromosome, but not co-localized, as expected from telomeric and centromeric markers (Fig. 1m–p). To continuously image chromosome dynamics in all neurons of live fish, a stable *tg(uas:EGFP-Terfa)* transgenic line was generated and crossed with *tg(HuC:Gal4)* zebrafish (Fig. 1q, r). Neurons in the telencephalon (Te), rhombencephalon (Rh), spinal cord (SC), and habenula (Hb), of 6-day post-fertilization (dpf) larvae were imaged during 9.5 min. Single-particle tracking (SPT) analysis^38^ was used to detect and quantify the motility of telomere trajectories (Supplementary Movie 1, Fig. 1s, t). While telomeres in the Rh, SC, and Hb neurons showed a similar volume of motion of 0.0069 ± 0.0002, 0.0066 ± 0.0003, and 0.0063 ± 0.0004 µm^3^, respectively, telomeres in Te neurons showed increased volume of motion (0.01 ± 0.0004 µm^3^, Supplementary Fig. 1a). Calculation of the mean square displacement (MSD) for single trajectories of all Te and Rh neurons demonstrated anomalous subdiffusion of the puncta (Fig. 1u), which is typical to chromosome diffusion^38^. Indeed, analysis of both telomeric and centromeric markers that are expressed in the same SC neurons (Fig. 1m–p, Supplementary Movie 2) showed similar dynamics (Supplementary Fig. 1b), verifying that the puncta mark chromosomes. Altogether, these results transfer in vitro chromatin experiments to whole organisms, and demonstrate the capability of monitoring chromosome dynamics in live larvae.

### Sleep increases chromosome dynamics in neurons

**Fig. 2:**
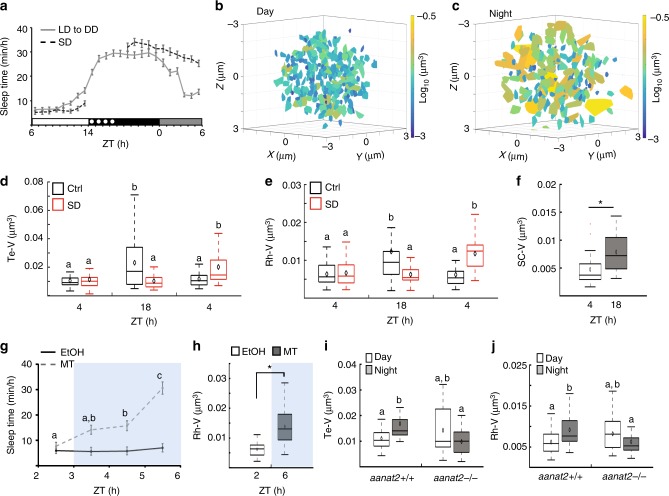
Sleep increases chromosome dynamics in neurons. **a** Recording of sleep was performed in 6 dpf control or sleep-deprived larvae under an 14 h light/10 h dark cycle following by constant darkness (LD to DD, control: *n* = 119 larvae, sleep deprivation (SD): *n* = 96 larvae). **b**, **c** The area scanned by all chromosomes from all imaged Te neurons during 9.5 min (day: *n* = 26 cells; night: *n* = 29 cells). Color was coded according to the levels of volume of motion. **d**–**f** Volume of chromosome dynamics over 9.5 min per cell (EGFP-Terfa in **d** and **e**, EGFP-Cenpa in **f**). **d** Te neurons, ctrl: day (*n* = 26 cells), night (*n* = 29 cells), subjective day (*n* = 25 cells). SD: day (*n* = 26 cells), night (*n* = 35 cells), subjective day (*n* = 25 cells). *P* = 1.3 × 10^−7^, *F* = 17.54, degrees of freedom = 2. **e** Rh neurons, ctrl: day (*n* = 23 cells), night (*n* = 30 cells), subjective day (*n* = 35 cells). SD: day (*n* = 27 cells), night (*n* = 25 cells), subjective day (*n* = 26 cells). *P* = 7 × 10^−6^, *F* = 12.82, degrees of freedom = 2, determined by two-way ANOVA followed by Tukey test. **f** SC neurons: day (*n* = 33 cells), night (*n* = 33 cells). *P = 0.0001, determined by two-tailed *t*-test: two samples assuming unequal variance. Red crosses indicate outliers. **g**, **h** Monitoring sleep and chromosome dynamics under melatonin (MT) treatment. Blue background represents time under treatment. **g** Pretreated and EtOH/MT-treated larvae (EtOH: *n* = 48; MT: *n* = 48 larvae). *P* = 7.4 × 10^−4^, *F* = 14.3, degrees of freedom = 3, determined by two-way ANOVA followed by Tukey test. Values are presented as means ± SEM. **h** Pre-treated cells (*n* = 22) and MT-treated cells (*n* = 25). *P = 7 × 10^−6^, determined by two-tailed *t*-test: two samples assuming unequal variance. **i** Te neurons, *aanat2* +/+ : day (*n* = 16 cells), night (*n* = 21 cells). *aanat2*−/−: day (*n* = 14 cells), night (*n* = 32 cells). *P* = 1.7 × 10^−3^, *F* = 12.26, degrees of freedom = 1. **j** Rh neurons, *aanat2*+/+ : day (*n* = 24 cells), night (*n* = 24 cells). *aanat2*−/−: day (*n* = 19 cells), night (*n* = 30 cells). *P* = 7.6 × 10^−4^, *F* = 10.42, degrees of freedom = 1, determined by two-way ANOVA followed by Tukey test. Boxplots: black diamond represents the mean, boxes indicate the median and the 25th-to-75th percentiles, whiskers extend to the most extreme data points. Letters or asterisks indicate significant differences. ZT zeitgeber time

In the diurnal zebrafish larvae, sleep is regulated by circadian and homeostatic processes and is defined as at least 1 min of immobility accompanied by an increased arousal threshold^15,39^. Continuous video tracking of behavior showed that, as is the case in WT larvae^40^, 6 dpf *tg(HuC:Gal4)*/*tg(uas:EGFP-Terfa)* larvae sleep more during the night, under a 14 h light/10 h dark to constant darkness (LD to DD) cycle (day: 8.6 ± 0.7 min/h; night: 28.05 ± 1.2 min/h; subjective day: 17.8 ± 1.01 min/h; Fig. 2a). Moreover, analysis of larval activity specifically during the wake bouts showed that the average time of activity is reduced during the night compared to the day (day: 5.6 ± 0.25 s/min; night: 1.6 ± 0.06 s/min, Supplementary Fig. 2). The behavioral immobility and reduced sensory input during sleep may favor behavioral-state-dependent cellular processes in neurons. Alterations in chromatin structure can affect a variety of nuclear processes, including genome stability, transcription, DNA repair, chromosome segregation, and condensation^24,37^. To test whether chromosome dynamics change between day and night, single nuclei were imaged in Te and Rh neurons of 6 dpf *tg(HuC:Gal4)*/*tg(uas:EGFP-Terfa)* larvae during the day [zeitgeber time 4 (ZT4)], night (ZT18), and the following subjective day (ZT4, Fig. 2a). Rh and Te neurons were selected because these regions regulate locomotor activity and cognition, and because these neurons exhibit standard and high levels of chromosome dynamics, respectively (Supplementary Fig. 1a). Remarkably, the time-lapse imaging of telomere markers showed that chromosome dynamics increased by approximately two-fold during nighttime sleep in both brain regions (average volume of motion, day: Rh—0.006 ± 0.0006 µm^3^, Te—0.01 ± 0.0008 µm^3^; night: Rh—0.012 ± 0.002 µm^3^, Te—0.023 ± 0.003 µm^3^; subjective day: Rh—0.006 ± 0.0005 µm^3^, Te—0.011 ± 0.001 µm^3^, Fig. 2b–e, Supplementary Movie 3, 4). Using centromeric markers, similar changes in chromosome dynamics were visualized in SC neurons (day: 0.0047 ± 0.0004 µm^3^; night: 0.0078 ± 0.0006 µm^3^, Fig. 2f, Supplementary Movie 2). In order to differentiate between sleep and circadian effect, the 6 dpf *tg(HuC:Gal4)*/*tg(uas:EGFP-Terfa)* larvae were sleep-deprived for 4 h during the night, and behavioral sleep rebound was observed during the following subjective day (ZT4; LD to DD: 12.4 ± 0.8 min/h; SD: 27.5 ± 1.3 min/h, Fig. 2a). Immediately following SD, chromosome dynamics were reduced by approximately two-fold (Rh—0.0062 ± 0.0005 µm^3^, Te—0.01 ± 0.0009 µm^3^, Fig. 2d, e) compared with the levels observed during the night in the control group, and were similar to the levels observed during the day. On the following day, after 10 h of recovery, when the sleep-deprived larvae demonstrated sleep rebound (Fig. 2a), chromosome dynamics increased by approximately two-fold (Rh—0.011 ± 0.0008 µm^3^, Te—0.02 ± 0.002 µm^3^, Fig. 2d, e) in the sleep-deprived larvae, and reached the levels observed during nighttime sleep in the sibling control larvae. These results show that sleep increases chromosome dynamics in a homeostatic-dependent manner.

To further validate the regulation of chromosome dynamics by the sleep state, the *tg(HuC:Gal4)*/*tg(uas:EGFP-Terfa)* larvae were treated during the day with melatonin, which is a strong sleep-promoting hormone in the diurnal zebrafish^14,41^. Sleep time and chromosome dynamics were monitored prior to and during melatonin treatment. As expected, while ethanol (EtOH) administration did not affect sleep time, under 3 h of melatonin treatment, sleep increased in melatonin-treated larvae (prior to melatonin treatment: 7.7 ± 1.5 min/h; 3 h following melatonin treatment: 30.7 ± 2.4 min/h, Fig. 2g). In accordance, melatonin-derived sleep increases chromosome dynamics (prior to melatonin treatment: 0.006 ± 0.0005 µm^3^; during melatonin treatment: 0.013 ± 0.0013 µm^3^, Fig. 2h). Thus, sleep is sufficient to increase chromosome dynamics. In order to understand if sleep is not only sufficient but also necessary to increase chromosome dynamics, we crossed the *tg(HuC:Gal4)*/*tg(uas:EGFP-Terfa)* zebrafish with arylalkylamine N-acetyltransferase-2^42^ mutant zebrafish (*aanat2*−/−), which lack melatonin signaling. The *aanat2*−/− larvae exhibit reduced sleep time during the night, although their intrinsic molecular circadian clock is intact^43^. Imaging single neurons during day and night revealed that while chromosome dynamics increased in *aanat2*+/+ larvae during the night (day: Rh—0.006 ± 0.0006 µm^3^, Te—0.01 ± 0.001 µm^3^; night: Rh—0.009 ± 0.0009 µm^3^, Te—0.014 ± 0.0008 µm^3^, Fig. 2i, j), as was the case in WT larvae (Fig. 2d, e), it was reduced during the night in *aanat2*−/− (night: Rh—0.006 ± 0.0006 µm^3^, Te—0.009 ± 0.0009 µm^3^) compared with *aanat2*+/+ larvae. Thus, although the molecular circadian clock is intact, chromosome dynamics were similar in both day and night, which is in accordance with the reduced nighttime sleep in *aanat2*−/− larvae (Fig. 2i, j). These results show that chromosome dynamics in neurons are regulated by the behavioral sleep/wake state.

Sleep-dependent changes in chromosome dynamics may not be specific to neurons. To test whether these changes are also present in other cell types, we monitored chromosome dynamics in peripheral endothelial and Schwann cells. Chromosome dynamics were imaged during day and night in endothelial and Schwann cells using *tg(fli:Gal4)*/*tg(uas:EGFP-Terfa)* and *mbp:Gal4*-injected *tg(uas:EGFP-Terfa)* 6 dpf larvae, respectively (Supplementary Fig. 3a, b). Chromosome dynamics did not differ between day and night in both cell types (Supplementary Fig. 3c). These results show that sleep-dependent changes in chromosome dynamics that were observed in neurons, do not occur in endothelial or Schwann cells.

### Sleep reduces DSBs that are accumulated during wakefulness

**Fig. 3:**
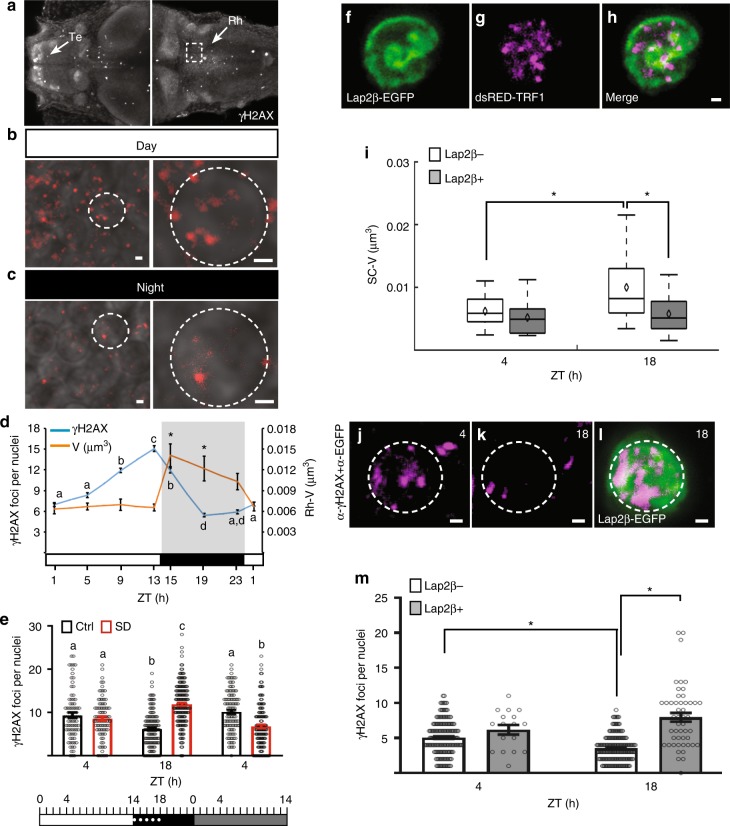
Chromosome dynamics are essential for reducing the number of accumulating DSBs. **a**–**c** Dorsal view of 6 dpf larvae stained with γH2AX. **a** Arrows indicate the telencephalon (Te) and rhombencephalon (Rh). Dashed box showing the Rh represents the area analyzed in **d** and **e**. Representative images from the Rh region during day (**b**) and night (**c**) are shown. Dashed circle indicates a single neuron. **d** The number of DSBs (*P* = 1 × 10^−16^, *F* = 118, degrees of freedom = 7) and chromosome dynamics (*P = 8 × 10^−7^, *F* = 7, degrees of freedom = 7) in single nuclei over 24 h, in 14 h light/10 h dark cycle. Determined by one-way ANOVA followed by a Tukey test. Lower case letters indicate significant changes between γH2AX groups. Asterisks indicate significant changes between chromosome dynamics groups. White and black horizontal bars represent light and dark periods, respectively. **e** Numbers of DSBs (dot plot, means ± SEM). Ctrl: day (*n* = 83 cells), night (*n* = 131 cells), subjective day (*n* = 105 cells). SD: day (*n* = 86 cells), night (*n* = 170 cells), subjective day (*n* = 135 cells). *P* = 1 × 10^−6^, *F* = 62.04, degrees of freedom = 2, determined by two-way ANOVA followed by a Tukey test. White, black, and gray rectangles represent day, night, and subjective day, respectively. Dotted white line represents the sleep deprivation (SD) period. **f**–**h** Co-expression of Lap2β-EGFP (**f**) and human telomeric markers (**g**) in SC-neuronal nucleus. **i** Volume of chromosome dynamics in control (Lap2β−, day: *n* = 25; night: *n* = 21 cells) and Lap2β-overexpressing cells (Lap2β+, day: *n* = 25; night: *n* = 19 cells). *P = 0.026, *F* = 5.1, degrees of freedom = 1, determined by two-way ANOVA followed by a Tukey test. **j**–**l** Representative images of double immunohistochemistry using α-γH2AX (magenta) and α-EGFP (green) in single SC neurons during day (**j**) and night (**k**, **l**). Dashed circle indicates a single nucleus. **m** The number of DSBs (dot plot, means ± SEM) in control (Lap2β−, day: *n* = 177; night: *n* = 177 cells) and Lap2β-overexpressing cells (Lap2β+, day: *n* = 17; night: *n* = 49 cells). *P = 1 × 10^−6^, *F* = 18.8, degrees of freedom = 1, determined by two-way ANOVA followed by a Tukey test. Zeitgeber time (ZT4)-day, ZT18-night. Boxplots: black diamond represents the mean, boxes indicate the median and the 25th-to-75th percentiles, whiskers extend to the most extreme data points. Letters or asterisks indicate significant differences. Scale bar = 1 µm

What is the physiological benefit of sleep-dependent chromosome dynamics? Since the genome can be hit by dozens of DSBs per day^33,44^, we speculated that sleep and increased chromosome dynamics are essential for the recovery from wakefulness-derived DNA damage. To test our hypothesis, the levels of DSBs and chromosome dynamics were monitored in Rh neurons during the 24-h sleep/wake cycle. The γH2AX marker, which is activated as part of the DNA damage response system^45^, was used to quantify DSBs during day and night in single cells (Fig. 3a–e). Whole head staining showed increased localization of γH2AX in the Te compared to other brain areas, such as the Rh (Fig. 3a). This DSB enrichment is correlated with the increased neuronal activity detected in the Te (Supplementary Fig. 4). In the 24-h experiment, we quantified DSBs in the Rh because it better represents the distribution of γH2AX in the entire CNS. During the day, the number of DSBs consistently increased, and peaked 1 h before darkness (15.1 ± 0.46 γH2AX foci, Fig. 3d). During the night, the number of DSBs dramatically decreased, reached the minimum levels at ZT19 (5.4 ± 0.24 γH2AX foci, Fig. 3d), and remained low until the beginning of the day. Concurrently, during the day, chromosome dynamics remained at similar low levels (ZT1: 0.0063 ± 0.0007; ZT5: 0.0067 ± 0.0005; ZT9: 0.007 ± 0.0008; ZT13: 0.0065 ± 0.0005 µm^3^, Fig. 3d). In contrast, following 1 h of darkness, chromosome dynamics increased by two-fold, and the high levels were maintained during the entire night (ZT15: 0.014 ± 0.0016; ZT19: 0.012 ± 0.0018; ZT23: 0.01 ± 0.0011 µm^3^, Fig. 3d). These results show that while chromosome dynamics keep constant low levels, DSB number accumulates during the day. During the night, following robust increase in chromosome dynamics, the number of DSBs was gradually reduced, showing that chromosome dynamics correlate with efficient reduction of DSBs during the night.

In order to distinguish between circadian and sleep effect on DSB levels, we quantified the number of DSBs in sleep-deprived larvae. In the control group, the number of γH2AX foci per cell in the Rh neurons decreased during the night and increased back to daytime levels on the following subjective day (day: 9.3 ± 0.61; night: 6.1 ± 0.36, subjective day: 10.16 ± 0.46, Fig. 3e). In contrast, following SD, the γH2AX foci number increased during the night (11.9 ± 0.37), and then, post-sleep recovery, was reduced on the following subjective day (6.76 ± 0.44, Fig. 3e). These experiments show that DSBs increased during wakefulness and decreased during sleep in neurons.

In order to test whether the amount of DSBs changed between day and night in other cell types, we performed immunostaining assays using both anti-γH2AX and anti-EGFP in *tg(fli:EGFP)* and tg(*mbp:EGFP)* 6 dpf larvae (Supplementary Fig. 3d-i). In contrast to the day/night changes observed in neurons, the number of DSBs was constantly low during day and night in endothelial and Schwann cells (Supplementary Fig. 3j), suggesting that wakefulness and sleep do not affect the levels of DSBs in these cells.

### Chromosome dynamics are necessary to reduce DSBs

In order to causally link chromosome dynamics and the efficient reduction in DNA damage, we manipulated chromosome dynamics by overexpressing the zebrafish Lamina-associated polypeptide 2 (Lap2β) in specific neurons. This protein physically interacts with lamins and anchors chromatin to the nuclear lamina in mammals and zebrafish^46,47^. In order to monitor chromosome dynamics and DSBs in Lap2β-overexpressing and control neurons, *pT2-uas:Lap2β-EGFP* and *pT2-uas:dsRED-TRF1* constructs were co-injected into *tg(HuC:Gal4)* embryos, and neurons that express either both Lap2β-EGFP and dsRED-TRF1 (Fig. 3f–h) or only dsRED-TRF1 in the SC were analyzed during day and night in 6 dpf larvae. As expected, chromosome dynamics increased during the night in the control dsRED-TRF1-expressing neurons (day: 0.006 ± 0.0004; night: 0.009 ± 0.001 µm^3^, Fig. 3i). In contrast, overexpression of Lap2β inhibited chromosome dynamics, specifically during the night, and the levels were similar during both day and night (day: 0.005 ± 0.0005; night: 0.005 ± 0.0007 µm^3^, Fig. 3i). Thus, the overexpression of Lap2β impedes the sleep-dependent increase of chromosome dynamics. Next, we measured the number of γH2AX foci in the control and in Lap2β-overexpressing neurons. As was found in the Rh (Fig. 3b–e), the number of γH2AX foci per neuron in the SC decreased by approximately 30% during the night (day: 5.1 ± 0.18; night: 3.5 ± 0.14, Fig. 3j, k, m). Remarkably, the number of γH2AX foci increased by 120% in Lap2β-overexpressing neurons compared with the control neurons during the night (7.95 ± 0.63 γH2AX foci, Fig. 3l, m). These results show that genetic inhibition of chromosome dynamics increases the number of DSBs specifically during nighttime sleep. Furthermore, these results suggest that sleep-dependent increase in chromosome dynamics is necessary to reduce DNA damage.

### Neuronal activity can reduce chromosome dynamics

**Fig. 4:**
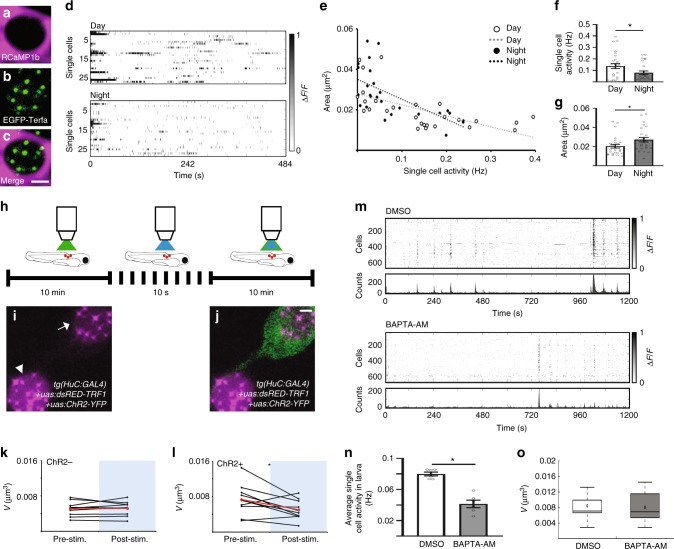
Neuronal excitation reduces chromosome dynamics in the Rh and SC. **a**–**c** Simultaneous 2D imaging of neuronal activity (magenta) and chromosome dynamics (green) in the Rh neurons. **d** Raster plot of Δ*F*/*F* of 27 and 26 RCaMP1b-expressing single cells during day and night, respectively. Grayscale: Δ*F*/*F* amplitude. **e** A moderate negative correlation between chromosome dynamics and neuronal activity during day and night (*R*_Day_ = −0.62, *R*_Night_ = −0.64, *R*_Total_ = −0.64), determined by Pearson correlation coefficient. The average single-cell activity (**f**, *P_activity_ *=* 0.035) and chromosome dynamics (**g**, *P_dynamics_ *=* 0.03) during day and night. Determined by two-tailed *t*-test: two samples assuming unequal variance. **h**–**l** Optogenetic stimulation of neuronal activity reduces chromosome dynamics. **h**–**j** The neurons expressed both dsRED-TRF1 and ChR2-YFP (arrow) or only dsRED-TRF1 (arrowhead). **k**, **l** Chromosome dynamics before and following the light stimuli (ChR2−, *n* = 9 cells; ChR2+, *n* = 10 cells). *P = 0.031, determined by two-tailed *t*-test: two-paired samples for means. Average change is marked by red line. **m**–**o** Inhibition of neuronal activity during the night does not affect chromosome dynamics. **m** Representative raster plots of Δ*F*/*F* in the Rh of 6 dpf *tg(HuC:GCaMP5)* larvae under DMSO (*n* = 6 larvae) or BAPTA-AM (*n* = 6 larvae) treatments. Grayscale: Δ*F*/*F* amplitude. Bottom: histogram of the Ca^2+^ transients of all cells. **n** Average spontaneous neuronal activity in single neurons of the Rh under either DMSO (*n* = 6 larvae) or BAPTA-AM (*n* = 6 larvae) treatment (ZT23). *P = 2 × 10^−4^, *t* = 2.3, degrees of freedom = 7, determined by two-tailed *t*-test: two samples assuming unequal variance. **o** Volume of chromosome dynamics during nighttime (ZT23) in DMSO (*n* = 13 cells) and BAPTA-AM (*n* = 11 cells)-treated larvae. Values are presented as boxplots and means (black diamonds) or as dot plots and means ± SEM. Boxplots indicate the median and the 25th-to-75th percentiles. The whiskers extend to the most extreme data points. ZT zeitgeber time. Scale bar = 2 µm

Which cellular processes induce DSBs during wakefulness, and how do they affect chromosome dynamics? In mammals, neuronal activity promotes the formation of DSBs^34,35^. In zebrafish, we found sleep-dependent changes in the levels of chromosome dynamics and DSBs in neurons (Figs. 2 and 3) but not in two other non-excitable cell types (Supplementary Fig. 3), and showed correlation between increased spontaneous neuronal activity and enriched expression of γH2AX in the Te (Fig. 3a, Supplementary Fig. 4). Therefore, since chromosome dynamics is required to reduce the number of DSBs we rationalized that the intensity and frequency of neuronal activity should affect chromosome dynamics within individual neurons. To study activity-dependent chromosome dynamics, a *tg(uas:RCaMP1b)* transgenic fish was generated and crossed with the *tg(HuC:Gal4)*/*tg(uas:EGFP-Terfa)* line. Then, spontaneous chromosome dynamics and neuronal activity were simultaneously imaged in one plane in single neurons within the Rh during day and night (Fig. 4a–c). The results showed moderate negative correlation between neuronal activity and chromosome dynamics (Fig. 4d–g). While the average frequency of single cell activity was reduced (day: 0.138 ± 0.022 Hz; night: 0.079 ± 0.015 Hz, Fig. 4d–f), the average chromosome dynamics increased during the night in Rh neurons (day: 0.02 ± 0.002 µm^2^; night: 0.027 ± 0.002 µm^2^, Fig. 4e, g). Taking into account that neurons in the Rh and SC regulate tail movement and locomotor activity^48^, which are markedly reduced during sleep, these results show that, at the single-cell level, chromosome dynamics increased in resting cells during the night.

In order to causally test the effect of neuronal activity on chromosome dynamics, we used optogenetics and transiently expressed the neural-activating cation channel channelrhodopsin-2 (ChR2-YFP)^49^ and the telomere marker dsRED-TRF1 in neurons of the SC (Fig. 4h–j), which showed similar chromosome dynamics and spontaneous neuronal activity as in Rh neurons (Supplementary Fig. 1a, Supplementary Fig. 4). Chromosome dynamics were quantified in single cells that did or did not express the ChR2-YFP before and following blue light stimulation during daytime wakefulness (Fig. 4h–j). While chromosome dynamics did not change post-stimulation in dsRED-TRF1-expressing cells (Fig. 4k), they were reduced in stimulated dsRED-TRF1+ChR2-YFP-expressing cells (pre-stimulation: 0.0073 ± 0.001 µm^3^; post-stimulation: 0.005 ± 0.0007 µm^3^, Fig. 4l). Thus, neuronal activity reduces chromosome dynamics in a single active cell under spontaneous conditions and optogenetic manipulation.

Neuronal activity could affect chromosome dynamics via the formation of DSBs. In order to uncouple neuronal activity from DNA damage, neuronal activity was inhibited specifically during the night, when the DSB level is low. The Ca^+2^ chelator BAPTA-AM, which lowers intracellular Ca^+2^ levels, was used. To validate the efficiency of BAPTA-AM in Rh neurons, we quantified spontaneous neuronal activity in BAPTA-AM-treated and control 6 dpf *tg(HuC:GCaMP5)* larvae. As expected, the average activity of a single Rh neuron was reduced by two-fold in BAPTA-AM-treated larvae compared with the control group (DMSO: 0.08 ± 0.002 Hz; BAPTA-AM: 0.04 ± 0.004 Hz, Fig. 4m, n). In these neurons, chromosome dynamics were monitored at the end of the night (ZT23), when the DNA damage is low (Fig. 3d). Inhibition of neuronal activity did not affect chromosome dynamics, which remain high in both BAPTA-AM-treated and control larvae (DMSO—0.0084 ± 0.001, BAPTA-AM—0.008 ± 0.001 µm^3^, Fig. 4o). These results indicate that neuronal activity is not an essential regulator of chromosome dynamics; however, it can reduce chromosome dynamics, possibly via the induction of DSBs.

### Sleep and chromosome dynamics increased following induction of DSBs

**Fig. 5:**
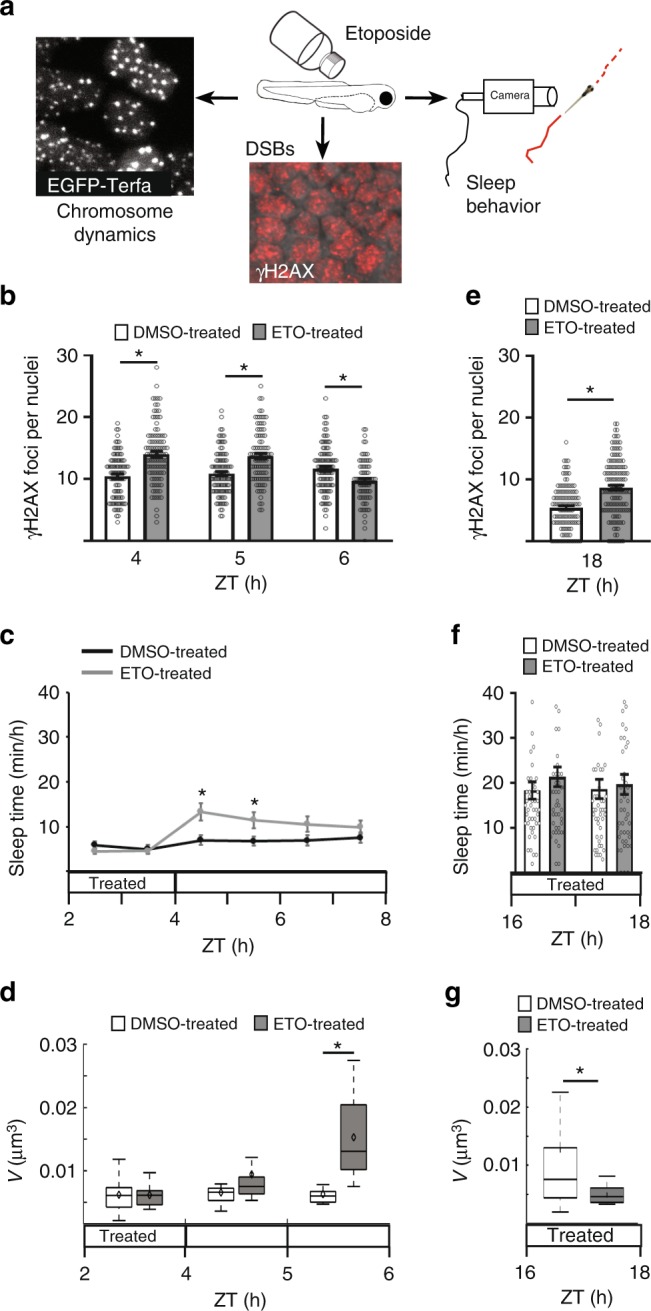
Sleep and chromosome dynamics increased following induction of DSBs. **a** Larvae were treated with etoposide (ETO) during the day (ZT2–4, **b**–**d**) and night (ZT16–18, **e**–**g**), and DSBs, chromosome dynamics, and sleep were quantified. **b** Number of γH2AX foci in single nuclei. During treatment (DMSO: *n* = 93 cells; ETO: *n* = 99 cells, *P = 2.2 × 10^−8^), 1 h following treatment (DMSO: *n* = 102 cells; ETO: *n* = 89 cells, *P = 2.2 × 10^−6^), 2 h following treatment (DMSO: *n* = 98 cells; ETO: *n* = 73 cells, *P = 6.8 × 10^−4^). **c** Total sleep time. DMSO-treated (*n* = 84 larvae), ETO-treated (*n* = 78 larvae), *P*_ZT2–3_ = 0.18, *P*_ZT3–4_ = 0.71. Post-treatment: DMSO-treated (*n* = 84 larvae), ETO-treated (*n* = 78 larvae), *P_ZT4–5_ = 0.0035, *P_ZT5–6_ = 0.02, *P*_ZT6–7_ = 0.07, *P*_ZT7–8_ = 0.2. **d** Chromosome dynamics in neurons. During treatment (DMSO: *n* = 22 cells; ETO: *n* = 11 cells), 1 h following treatment (DMSO: *n* = 17 cells; ETO: *n* = 13 cells), 2 h following treatment (DMSO: *n* = 11 cells; ETO: *n* = 16 cells, *P = 3.8 × 10^−5^). Red crosses indicate outliers. **e**–**g** Number of γH2AX foci in single neuronal nuclei (**e**, DMSO: *n* = 118; ETO: *n* = 138 cells, *P = 1.65 × 10^−9^), total sleep time (**f**, DMSO: *n* = 48; ETO: *n* = 48 larvae) and chromosome dynamics (**g**, DMSO: *n* = 22; ETO: *n* = 14 cells, *P = 0.02) in DMSO- and ETO-treated larvae during the night. Significant differences of all experiments were determined by two-tailed *t*-test: two samples assuming unequal variance. Values are presented as boxplots and means (black diamonds) or as dot plots and means ± SEM. Boxplots indicate the median and the 25th-to-75th percentiles. The whiskers extend to the most extreme data points. ZT zeitgeber time. Scale bar = 1 µm

Neuronal activity is only one of several processes that can induce DSBs during wakefulness. The causes for DNA damage are diverse and include intrinsic and extrinsic factors^33^. In order to simulate the effect of these different factors, we used etoposide (ETO), which induces DSBs^45^, and the number of γH2AX foci, chromosome dynamics, and sleep time were monitored during the day (Fig. 5a–d). As expected, after 2 h under ETO treatment (ZT2–4), the γH2AX foci number increased (DMSO: 10.44 ± 0.37; ETO: 14.01 ± 0.47, Fig. 5b). Furthermore, sleep time (Fig. 5c) and chromosome dynamics (Fig. 5d) did not change under ETO treatment. However, 1 h following ETO withdrawal, sleep time increased in ETO-treated larvae (DMSO: 6.95 ± 1.1 min/h; ETO: 13.28 ± 1.8 min/h, Fig. 5c), while DSB levels remained high (DMSO: 10.87 ± 0.35; ETO: 13.7 ± 0.46 γH2AX foci, Fig. 5b) and chromosome dynamics remained low (DMSO: 0.0065 ± 0.0004; ETO: 0.0094 ± 0.001 µm^3^, Fig. 5d). Notably, following 2 h of recovery from the ETO treatment, sleep time remained high (DMSO: 6.71 ± 1 min/h; ETO: 11.4 ± 1.8 min/h, Fig. 5c), and chromosome dynamics increased by approximately two-fold (DMSO: 0.0062 ± 0.0005; ETO: 0.015 ± 0.0017 µm^3^, Fig. 5d), accompanied by a reduction of the number of DSBs (9.65 ± 0.42 γH2AX foci, Fig. 5b). In order to examine how the formation of DSBs will affect sleep time and chromosome dynamics during the night, we exposed the larvae to ETO for 2 h (ZT16–18). Similar to the results obtained during the day, under ETO treatment, the γH2AX foci number increased by 60% (DMSO: 5.4 ± 0.33; ETO: 8.66 ± 0.4, Fig. 5e) and sleep time did not change (Fig. 5f). However, chromosome dynamics decreased by approximately two-fold (DMSO: 0.0126 ± 0.003; ETO: 0.0049 ± 0.0004 µm^3^, Fig. 5g). These results suggest that while chromosome dynamics are low during the formation of DSBs, the accumulation of DSBs during wakefulness triggers sleep, which increases chromosome dynamics and eventually reduces the number of DSBs.

## Discussion

**Fig. 6:**
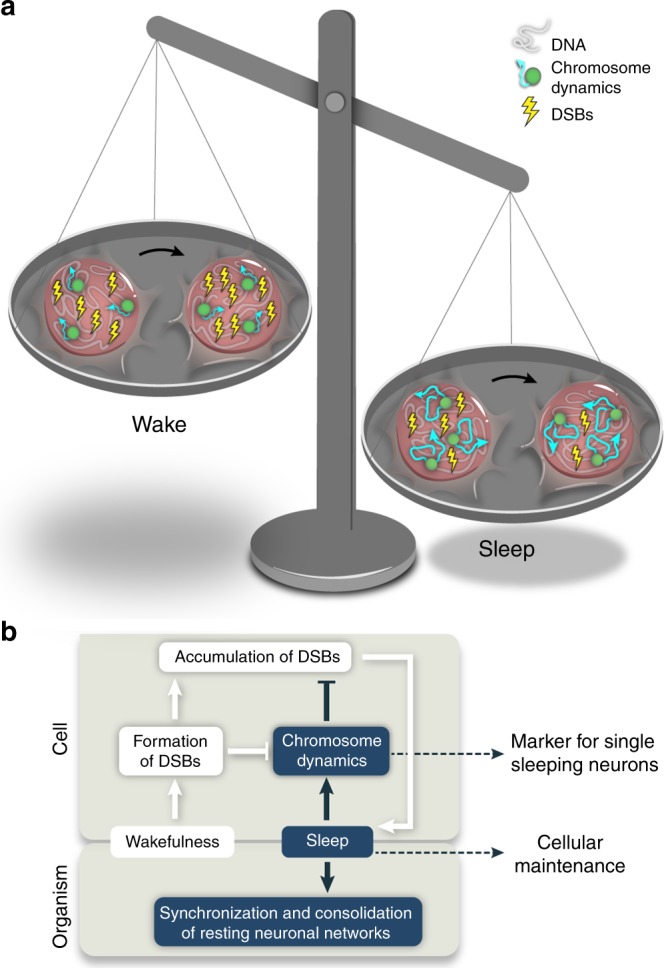
A proposed function for sleep. **a** During wakefulness, chromosome dynamics are low and the number of DSBs is accumulated in neurons. The beneficial role of sleep is to increase the chromosome dynamics that are essential for the efficient reduction of the number of DSBs in single neurons. **b** DSBs are increased in the nucleus during wakefulness and are formed by intrinsic and extrinsic factors, such as neuronal activity, irradiation, and oxidative stress, when chromosome dynamics are low. At a given threshold, accumulations of DSBs in multiple neuronal networks can trigger sleep, which increases chromosome dynamics that are necessary for the reduction of DNA damage. This mechanism suggests that chromosome dynamics can define single sleeping neurons, and that one of the functions of sleep is nuclear maintenance

The beneficial role of sleep in simple neuronal networks and in the complex brain is a mystery^50,51^, and it is unclear if and why a single neuron requires sleep. The methods developed in this work enable the study of sleep-dependent nuclear processes in single cells of live animals. We visualized 3D motions of individual chromosomes, DSBs, neuronal activity and behavior in live zebrafish, and directly linked the physiology of single neurons to the entire organism during sleep and wakefulness. Genetic and pharmacological manipulation of the melatonin signaling, as well as SD experiments, revealed that chromosome dynamics increased about two-fold during sleep in neurons. These sleep-dependent changes were not observed in two other non-excitable cell types. In neurons, DSBs accumulate during wakefulness and SD, when chromosome dynamics are low. Imaging of spontaneous neuronal activity, single-cell optogenetic and pharmacological experiments indicated that DSB formation by neuronal activity and other factors, can reduce chromosome dynamics. In turn, sleep benefits the brain because it increases chromosome dynamics, which are essential for the efficient reduction of the number of DSBs (Fig. 6).

Why do chromosome dynamics increase during sleep? The dynamic organization of the genome is often altered in diseases^52^, and changes in chromatin dynamics have been shown to regulate key nuclear processes, including epigenetics, DNA damage and repair, transcription, and enhancer–promoter interaction. For example, transcriptional activation by enhancers and promoters is associated with an increase in their loci mobility in live embryonic stem cells^53^. However, although across the sleep–wake cycle global gene expression in the brain is dynamically regulated, the expression of the majority of genes peaks during wakefulness^9,54^, whereas chromosome dynamics increase during sleep. Thus, regulation of the expression of specific genes by chromosome dynamics should be explored. Alternatively, we propose that increased chromosome dynamics enhance the efficiency of DSB elimination during sleep. Notably, although chromosome mobility increases following induction of DSBs, the increase appears to require a threshold level of DNA damage in yeast^55^. In agreement with that, we show that continuous wakefulness inhibits chromosome dynamics, thus preventing the efficient repair of DSBs. We propose that at a given threshold level of DNA damage, sleep is triggered in order to induce chromosome dynamics, which enable the cell to balance between DNA damage and repair (Fig. 6a). Inhibition of a sleep-dependent increase in chromosome dynamics, as demonstrated in the Lap2β-overexpressing neurons, can lead to accumulation of DSBs and potentially to cell death. This mechanism can perhaps explain why prolonged sleep deprivation can be lethal^6^, and why aging and neurodegenerative disorders are associated with both abnormal sleep^56^ and increased mutations in the genome of human neurons^57^.

Sleep is typically monitored at the network and whole-organism levels. However, by overcoming a technology barrier, this work determined the effect of sleep on the physiology of single neurons in the context of a live organism. At the cellular level, our results show that a single DNA damaged neuron, but not an endothelial or Schwann cell, requires relatively long periods of sleep in order to increase chromosome dynamics and eliminate the accumulation of DSBs. Indeed, in mammalian cells and embryonic zebrafish cells, clearance of γH2AX-stained foci typically takes several hours^58–60^. These nuclear processes are not efficient during prolonged wakefulness, possibly because all cell resources and energy are involved in neuronal function^61^. In non-neuronal cell types, such as endothelial and Schwann cells, repair of DSBs may occur in a cell-autonomous manner, which does not affect the behavior of the whole organism. The molecular mechanisms that regulate chromosome dynamics in neurons during sleep and wake require further investigation. Lap2β and lamin A are two potential regulators^38^; however, the involvement of other factors, such as proteins of the linker of the nucleoskeleton and cytoskeleton (LINC) complex^26^, cannot be excluded.

At the whole-organism level, an important question is how wake and sleep states affect these cellular processes. We propose that sleep behavior synchronizes and consolidates local sleep in neuronal networks in order to perform essential nuclear maintenance that requires prolonged periods of reduced body movement and responsiveness to external stimuli (Fig. 6b). This is essential because desynchronized networks, where individual neurons reach the rest threshold at various time points, would have disabled the behavioral performance of the animals. Nevertheless, this explanation may not settle with the relatively high global brain activity during sleep that is only slightly lower than during wakefulness in zebrafish^62^. Likewise, in mammals, based on EEG measurements in the cortex, global activity during rapid eye movement (REM) sleep is almost similar to the activity during wakefulness^63^. Yet, these studies and experimental approaches focused on average neuronal activity across large brain regions, such as the cortex, while here, we determined the effect of sleep on single neurons, and can differentiate single wake- versus sleep-active neurons. Similarly, in mice, microendoscopic calcium imaging of single cells in the dorsal pons showed that wake-active GABAergic neurons are significantly more active during wakefulness than REM and non-rapid eye movement (NREM) sleep^64^. In the zebrafish rhombencephalon, we identified mainly wake-active neurons, suggesting that, at least in the hindbrain but also likely in additional deep brain regions, neurons reduce their activity during sleep. Since, to date, different sleep stages were not detected in fish, future works in mammals are expected to enhance understanding of the correlation between EEG, neuronal activity, and chromosome dynamics in specific neuronal populations during wakefulness, REM, and NREM sleep.

What is the conserved function of sleep? How is it regulated? And how can sleep be defined at the cellular level? Studying sleep across phylogeny can provide key insights into these fundamental questions^50^. Using non-mammalian models, several sleep genes were identified^1,65^, and sleep-dependent structural synaptic plasticity was visualized in worms, flies, and zebrafish^66–68^. However, conserved functions and cellular markers were not yet identified. We suggest that in zebrafish, sleep is beneficial to the brain because single neurons can increase chromosome dynamics and perform nuclear maintenance during a consolidated offline period. Future studies on additional vertebrate and invertebrate multicellular animals, ranging from cnidarians to mammals, can establish chromosome dynamics as an evolutionarily conserved cellular sleep marker. This marker may be used to define sleeping single neurons in a live mounted animal even under the microscope, where behavioral criteria are insufficient. Altogether, we postulate that sleep behavior has evolved in order to regulate rest bouts of functionally linked single neurons, thus enabling coordinated nuclear maintenance.

## Methods

### Zebrafish husbandry and transgenic lines

Adult zebrafish (male and female) were raised and maintained in fully automated zebrafish housing systems (Aquazone, Israel; temperature 28 ± 0.5 °C, pH 7.0, conductivity 500 μS) under 14 h light/10 h dark cycles, and fed twice a day. Embryos were produced by natural spawning and raised in egg-water containing methylene blue (0.3 ppm) in a light-controlled incubator at 28 ± 0.5 °C and under 14 h light/10 h dark cycle. All experiments were done at the larval stages and specific age is indicated at the manuscript. The transgenic lines *tg(uas:EGFP-Terfa)* and *tg(uas:RCaMP1b)* were generated using the Tol2 system^69^. The transgenic lines *tg(mbp:EGFP), tg(HuC:Gal4), tg(fli:Gal4)* and *tg(fli:EGFP)*, as well as *tg(HuC:GCaMP5)* were kindly provided by Cheol-Hee Kim (Chungnam National University Daejeon, Korea), Bettina Schmid (Ludwig Maximilian University of Munich, Germany), Karina Yaniv (Weizmann Institute of Science, Israel), and German Sumbre (Institute de Biologie-Ecole Normale Superieure, France), respectively. The *aanat2*−/− mutant was kindly provided by David Prober (California Institute of Technology, CA, USA). All animal protocols were reviewed and approved by the Bar-Ilan University Bioethics Committee.

### DNA constructs

In order to prepare constructs of chromosome markers, the coding sequences of *terfa* (NM_173243.2) and *cenpa* (NM_001164240.1) were amplified using the following primers: *Terfa*: 5′-ccaaccggtatgagcgacaaaccctgc-3′ and 5′-ccaatcgattcagaccatcttgagcttgac-3′; *Cenpa*: 5′-ccaaccggtatgcctcgccatacatcg-3′ and 5′-ccaatcgatttacatgtgttcaacgcctctg-3′. The EGFP coding sequence was amplified with 5′-accgaattcaccatggctccaaagaagaagcgtaaggtaatggtgagcaagggcgaggagc-3′ and 5′-ccaaccggtcttgtacagctcgtccatgcc-3′ primers, the forward primer includes *nls* sequence (underlined). Triple ligation of *EcoRI*/*ClaI*-digested *pT2-uas:MCS* vector, *EcoRI*/*AgeI-*digested *egfp*, and *AgeI*/*ClaI-*digested *terfa* or *AgeI*/*ClaI-*digested *cenpa* were performed. *pT2-uas:dsRED-TRF1* was generated by amplifying dsRED-TRF1 flanked by *EcoRI*/*ClaI* from *cmv:dsRED-TRF1* vector (primers: 5′-accgaattcatgaagcttgcctcctccgag-3′ and 5′-ccaatcgattcagtcttcgctgtctgagga-3′), and cloning into *EcoRI*/*ClaI*-digested *pT2-uas:MCS* vector.

The human TRF1 DNA binding site contains three helices located at the C-terminus of the protein^70^. BLAST analysis of the human TRF1 and zebrafish Terfa revealed homology (68%) of these motifs. To delete the DNA binding site of Terfa, *pT2-uas:EGFP-Terfa del* construct was generated. The *pT2-uas:EGFP-Terfa* construct was amplified using the primers 5′-ggtgtactttttagctggagctgtagaagt-3′ and 5′-tgaatcgatgatgatccagacatgataaga-3′, and ligated using T4-ligase (NEB, Ipswich, MA, USA), which resulted in the deletion of 162 bp in the C-terminus and truncated protein.

In order to prepare the *pT2-mbp:Gal4* construct, approximately 1.9 Kb of the *mbp* promoter (AY860977) was amplified using the primers 5′-ccggggcccataataacaatcccaactc-3′ and 5′-ccgaccggtgtagtccttctccgctca-3′. The PCR product was digested with *ApaI* and *AgeI* restriction enzymes and cloned into *ApaI*- and *AgeI*-digested *pT2-hcrt:Gal4* vector replacing the *hcrt* promoter.

In order to prepare the *pT2-uas:RCaMP1b* construct, *RCaMP1b* coding sequence was amplified from *pGP-CMV-NES-jRCaMP1b* (gift from Douglas Kim, Addgene plasmid # 63136)^71^, using the 5′-ctcagatctcgccaccatgctgcaga-3′ and 5′-ttactcgaggcggccgcctacttcgctgtc-3′ primers. The PCR product was digested with *BglII* and *XhoI* restriction enzymes and cloned into *BglII* and *XhoI* digested *pT2-uas:MCS* vector. The *uas:ChR2-YFP* construct was kindly provided by Prof. Philippe Mourrain (Stanford University, USA).

In order to prepare the *pT2-uas:Lap2β-EGFP* construct, the coding sequences of *lap2β* (NM_001141787.1) were amplified using the primers 5′-acacgaattcaccatggctccaaagaagaagcgtaaggtaatgtcggaatttctggaga-3′ and 5′-gttgaattcttgctggtactgtcatctgtgccgctc-3′; the forward primer includes the *nls* sequence (underlined). The PCR product was digested by *EcoRI* restriction enzyme and cloned into *EcoRI*-digested *pT2-uas:EGFP* vector.

### Imaging

All imaging experiments were conducted on 6–7 dpf larvae. Larvae were mounted with low-melting-point agarose 2.0%, and paralyzed in 0.3 mg/ml pancuronium bromide (P1918, Sigma-Aldrich, St. Louis, MO, USA). Imaging was performed using a Zeiss LSM710 upright confocal/two-photon microscope (Zeiss, Oberkochen, Germany) with ×63, 1.0 NA objective. In order to standardize the optimal conditions for single chromosome imaging, we imaged the motion of single chromosomes using either 2-photon or confocal microscopes. Using either microscope, we observed changes in chromosome dynamics during day and night (2-photon: day—0.013 ± 0.002, night—0.025 ± 0.01; confocal: day—0.009 ± 0.002, night—0.027 ± 0.008; *n* = 5 Te neurons for each group). Imaging chromosome dynamics using Mai-Tai 2-photon laser (Spectra-Physics, Santa Clara, CA, USA), tuned to 920 nm excitation wavelength, resulted in relatively low signal-to-noise ratio. In contrast, imaging of chromosomes using the confocal microscope equipped with argon laser (488 nm) resulted in an efficient signal-to-noise ratio. This is because high-magnification confocal imaging capture larger focal plan that limits loss of particle trajectories in the *z*-axis. Therefore, in chromosome-dynamic experiments, we used a confocal microscope with the following parameters: image resolution of 256 × 256, 50 cycles of 30 planes, speed of 242.04 ms per plane. One plane size was typically 16.8 × 16.8 × 1.3 µm, with intervals of 0.3 µm between planes in all Z stacks.

In simultaneous confocal imaging of RCaMP1b and EGFP-Terfa (lasers: argon 488 nm, DPSS 561 nm), the following parameters were used: image resolution of 256 × 256, scanning time of 8 min at 4.13 Hz on 16.8 × 16.8 μm single plane. In *HuC-*driven GCaMP5 imaging, 6 dpf *tg(HuC:GCaMP5)* larvae were mounted, and GCaMP5 signal was monitored using a Mai-Tai 2-photon laser, tuned to 920 nm, with a ×20 1.0 NA objective. Scanning was performed at a single plane for 20 min at 4.13 Hz on 140 × 140 μm in Te and SC neurons, and 170 × 170 μm in Rh neurons.

### Transient expression assays

Transient expression assays of the DNA constructs *pT2-uas:EGFP-Terfa-del*, *pT2-uas:dsRED-TRF1*, *pT2-uas:EGFP-Cenpa*, *uas:ChR2-YFP*, *pT2-uas:Lap2β-EGFP*, and *pT2-mbp:Gal4* were performed by microinjection of approximately 2 nl plasmid into one-cell-stage embryos, at a concentration of 30 ng/µl each, using a micromanipulator and PV830 Pneumatic PicoPump (World Precision Instruments, Sarasota, FL). Imaging of all transient experiments was performed in 6 dpf larvae.

### Optogenetic experiments

The expression vectors *pT2-uas:dsRED-TRF1* and *pT2-uas:ChR2-YFP* were co-injected into one-cell-stage *tg(HuC:Gal4)* embryos, and positive embryos were raised under 14 h light/10 h dark cycles. At 6 dpf (ZT4), dsRED-TRF1- or dsRED-TRF1+ChR2-YFP-positive neurons were imaged for 10 min with DPSS laser (561 nm). Then, we stimulated the neurons ten times with blue light pulses in 1-s intervals. Following the stimulation, imaging of the same individual neurons was repeated using both DPSS (561 nm) and argon (488 nm) lasers.

### Melatonin experiments

Individual 6 dpf *tg(HuC:Gal4)*/*tg(uas:EGFP-Terfa)* live larvae were treated with either 10 µM melatonin (M5250, Sigma-Aldrich, St. Louis, MO, USA) or 0.7% EtOH during daytime. Sleep behavior and chromosome dynamics were monitored in Rh neurons of the same larvae before and during treatment with each compound.

### BAPTA-AM experiments

Individual 6 dpf *tg(HuC:Gal4)*/*tg(uas:EGFP-Terfa)* and *tg(HuC:GCaMP5)* live larvae were treated with either 5 mM BAPTA-AM (A1076, Sigma-Aldrich, St. Louis, MO, USA) or 0.1% DMSO diluted in embryo water for 4 h during the night (ZT19–23). During the 4th hour of treatment, chromosome dynamics and neuronal activity were monitored in Rh neurons.

### Etoposide experiments

6 dpf *tg(HuC:Gal4)*/*tg(uas:EGFP-Terfa)* larvae were treated for 2 h with either 10 µM etoposide (E1383, Sigma-Aldrich, St. Louis, MO, USA) or 0.01% DMSO diluted in embryo water during day or night. Following treatment, the compound was removed, and the larvae recovered in fresh water. Three parameters were monitored: number of DSBs, chromosome dynamics, and sleep behavior. To quantify the number of DSBs and chromosome dynamics in Rh neurons, larvae were either fixed (for immunohistochemistry) or live-imaged, respectively, following the compound treatment and during the recovery period. In behavioral assays, sleep was monitored as described below.

### Behavioral assays

In order to monitor sleep, larvae were individually placed in 48-well plates containing either embryo water, melatonin, ETO, EtOH, or DMSO compound. Larva-containing plates were placed in the Noldus DanioVision tracking system (Noldus Information Technology, Wageningen, Netherlands) under either light or dark. Live video-tracking were conducted using the EthoVision XT 12 software (Noldus Information Technology, Wageningen, Netherlands) with the following parameters for detection: dynamic subtraction, subject is darker than background, dark contrast 16–60, current frame weight 1, subject size of minimum 1 pixel and maximum 125,000 pixels, subject contour turned off, video sample rate of 12.5 frames per second, and no pixel smoothing. Data analyses of sleep time were performed according to the threshold parameters: distance, 0.3 cm; time, stop velocity 0.59 cm/s, start velocity 0.6 cm/s^40^. SD was performed on freely swimming larvae in Petri dishes by 4 h of gentle, random, and unsynchronized manual, mechanical tapping^11^. Sleep was monitored continuously prior to and 1 h following SD.

### Immunohistochemistry assays

Larvae were fixed for 2 h in 4% paraformaldehyde (PFA) in PBS at 4 °C, and washed in PBS + 0.5% Triton. The larvae were then blocked with 10% fetal bovine serum diluted in PBS for 1 h at room temperature. After blocking, the larvae were incubated in primary antibody: rabbit anti-EGFP (SC-8334, Santa Cruz Biotechnology, Santa Cruz, CA), 1:250 dilution; mouse anti-γH2AX (GTX127342, GeneTex), 1:100 dilution, in blocking buffer overnight at 4 °C. On the following day, larvae were washed in PBS + 0.5% Triton and blocked for 1 h. Anti-GFP was detected with a donkey polyclonal secondary anti-rabbit IgG H&L (Alexa Fluor® 488, 2 mg/ml, ab150061, Abcam), and anti-γH2AX was detected with donkey anti-mouse IgG H&L (Alexa Fluor® 594) (2 mg/ml, ab150064, Abcam), in blocking buffer overnight at 4 °C. Next, larvae were washed in PBS + 0.5% Triton, fixed for 30 min in 4% PFA in PBS, and washed in PBS + 0.5% Triton.

### Data analysis

3D SPT analysis of a time-lapse image sequence was performed in two stages. First, images were analyzed using Imaris software (Bitplane AG, Zurich, Switzerland), and punctum coordinates were located in each frame using the following parameters: detected ellipsoids with XY diameter of 0.3–0.6 µm and Z diameter of 1.3 µm, “quality” filter type to detect as much puncta as possible, and the “autoregressive motion” algorithm, with maximum distance of 1.25 µm without filling gaps. We verified that there was no concatenation between different puncta, and only punctum trajectories that were continuously trackable during the entire imaging session were quantified. In cases where the entire nucleus, and consequently all chromosomes, rotated significantly in a similar direction, the cells were excluded from the analysis. Then, only a nucleus with at least four reliable tracks was analyzed using custom-made MATLAB software (Mathworks, Natick, MA, USA) for nucleus drift and rotation correction^38^. In single-plane imaging, analysis was performed in the 1st minute in order limit loss of punctum trajectory in the *z*-axis.

In calcium imaging analysis (RCaMP1b and GCaMP5), imaging movies were registered to remove drifts using the Template Matching plugin in ImageJ. Then, using the custom-made MATLAB (Mathworks, Natick, MA, USA) programs FindROI and ProcessCalciumData^72^, regions of interest (ROI) were selected, and DeltaFoF and raster plot were determined.

To quantify the number of DSBs, snapshot images of 18 × 18 µm were acquired and the number of γH2AX foci per single cell (~15 cells per image) was counted manually using Cell Counter plugin in ImageJ.

### Statistical information

All imaging experiments were performed independently at least three times, whereas at least three larvae were imaged in each independent imaging experiment. In all experiments, larvae were selected randomly. Number of larvae, cells or chromosomes, statistical tests and *P* values are stated in each figure legend. Differences between two categorical groups (ctrl/SD and day/night/subjective day, EtOH/melatonin and pre-/post-treatment, day/night and *aanat2*+/+/*aanat2*−/− or day/night and Lap2β+/Lap2β−) on continuous variables (chromosome dynamics, sleep, or γH2AX foci number) were determined by two-way ANOVA using Statistica software (TIBCO). Differences between more than two groups were determined using one-way ANOVA using Statistica software (TIBCO). Differences between two groups were determined by two-tailed *t*-test: two samples assuming unequal variance using Analysis ToolPac in Excel. Changes between paired samples in the optogenetic experiments were determined by two-tailed *t*-test: paired two samples for means using Analysis ToolPac in Excel. Chromosome dynamics and neuronal activity correlation were determined by Pearson correlation coefficient using the social science statistics calculator.

### Reporting summary

Further information on experimental design is available in the Nature Research Reporting Summary linked to this article.

## Supplementary information

Supplementary Information

Description of Additional Supplementary Files

Supplementary Movie 1

Supplementary Movie 2

Supplementary Movie 3

Supplementary Movie 4

Reporting Summary

## Data Availability

The data that support the findings of this study are available from the corresponding author upon request.
