# Supplementary material for: Sleep increases chromosome dynamics to enable reduction of accumulating DNA damage in single neurons
Source: Nat Commun. 2019 Mar 5;10:895. doi: 10.1038/s41467-019-08806-w (PMC6401120; doi:10.1038/s41467-019-08806-w)
Supplement: Supplementary file 1 — Supplementary Information [file 41467_2019_8806_MOESM1_ESM.pdf]

Supplementary Information

Sleep increases chromosome dynamics to enable reduction of accumulating DNA damage in single neurons

Zada et al.

Supplementary Figure 1

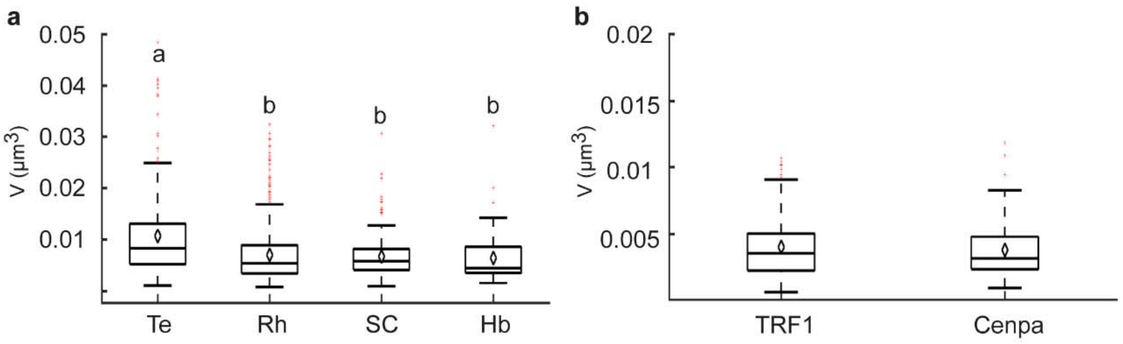

Chromosome dynamics in different brain regions. (**a**) Chromosome dynamics over 9.5 minutes in different CNS regions. Telencephalon (Te, n=340 chromosomes), rhombencephalon (Rh, n=475 chromosomes), spinal cord (SC, n=176 chromosomes), and habenula (Hb, n=95 chromosomes). $P=1.1\times10^{-16}$, $F=29.65$, degrees of freedom=3, determined by one-way ANOVA followed by a Tukey test. Letters indicate significant changes. (**b**) Telomere (TRF1, n=134 telomeres) and centromere (Cenpa, n=104 centromeres) dynamics over 9.5 minutes in SC neurons. $P=0.46$ determined by two-tailed *t*-test: two samples assuming unequal variance. Red crosses indicate outliers. Values are presented as boxplots and means (black diamonds). Boxplots indicate the median and the 25th-to-75th percentiles. The whiskers extend to the most extreme data points.

Supplementary Figure 2

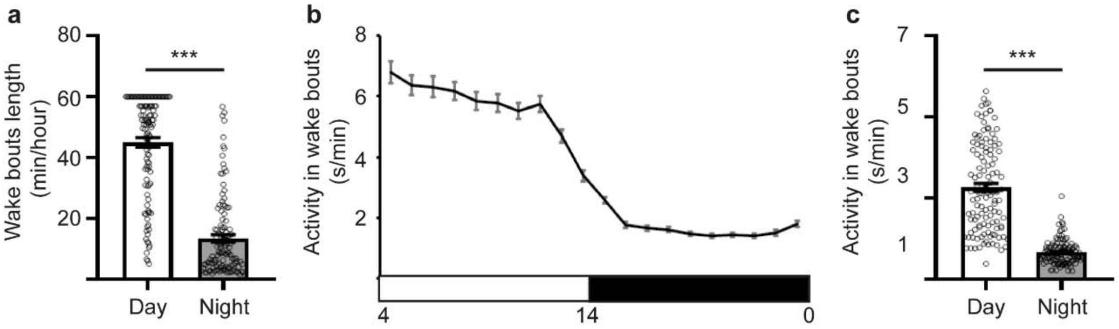

Reduced time of activity during wake bouts that occur during the night. (a) Average wake-bout length during day and night. ***P=2.8×10^{-39}. (**b**) Time of activity during wake bouts in 6 dpf larvae (n=119 larvae). White and dark bars represent day and night, respectively (**c**). Average time of activity during wake bouts. ***P=1.2×10^{-31}. Determined by two-tailed *t*-test: two samples assuming unequal variance. Values are presented as dot plots and means ± SEM.

Supplementary Figure 3

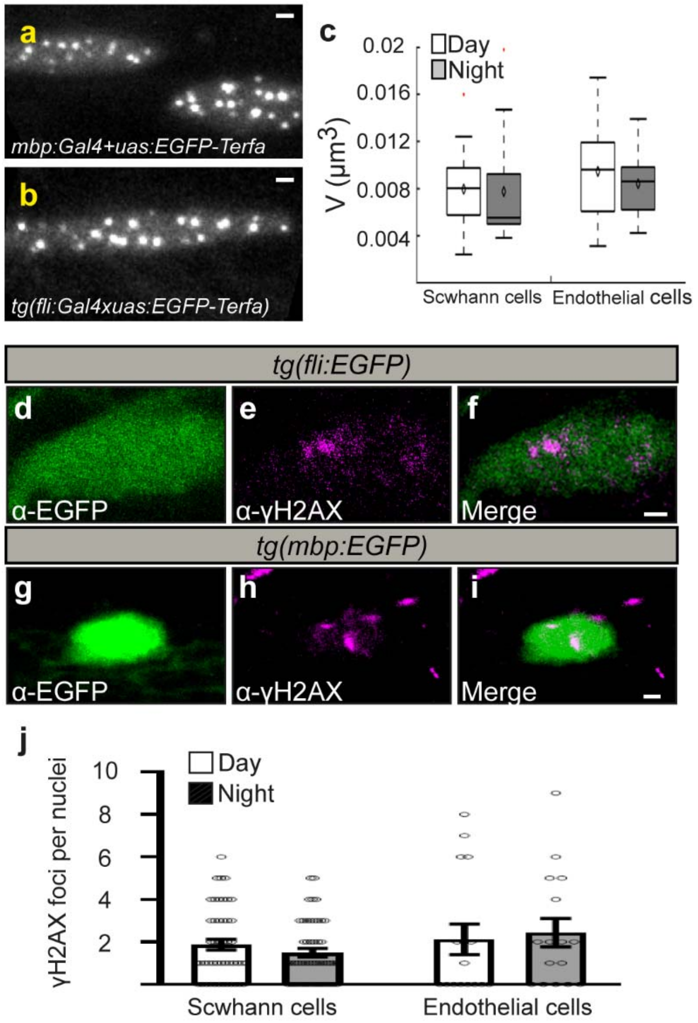

Chromosome dynamics and DSB levels are similar during day and night in Schwann and endothelial cells. Live 3D imaging of chromosome dynamics in the nucleus of Schwann cells (**a**) and endothelial cells (**b**) located in the trunk of 6 dpf larvae. (**c**) Boxplots and means (black diamonds) of the volume of chromosome dynamics over 9.5 min per cell. Schwann cells: Day (n=19 cells) and night (n=17 cells). Endothelial cells: Day (n=25 cells) and night (n=23 cells). Red crosses indicate outliers. Boxplots indicate the median and the 25th-to-75th percentiles. The whiskers extend to the most extreme data points. (**d-i**) Representative images of double immunohistochemistry using α-γH2AX (magenta) and α-

EGFP (green) in the trunk of *tg(fli:EGFP)* (**d**-**f**) and *tg(mbp:EGFP)* (**g-i**). (**j**) The number of γH2AX foci in single nuclei of Schwann (day: n=47; night: n=49 cells) and endothelial cells (day: n=16; night: n=16 cells) during day and night. Values are represented as dot plots and means ± SEM. Scale bar = 1 μm.

Supplementary Figure 4

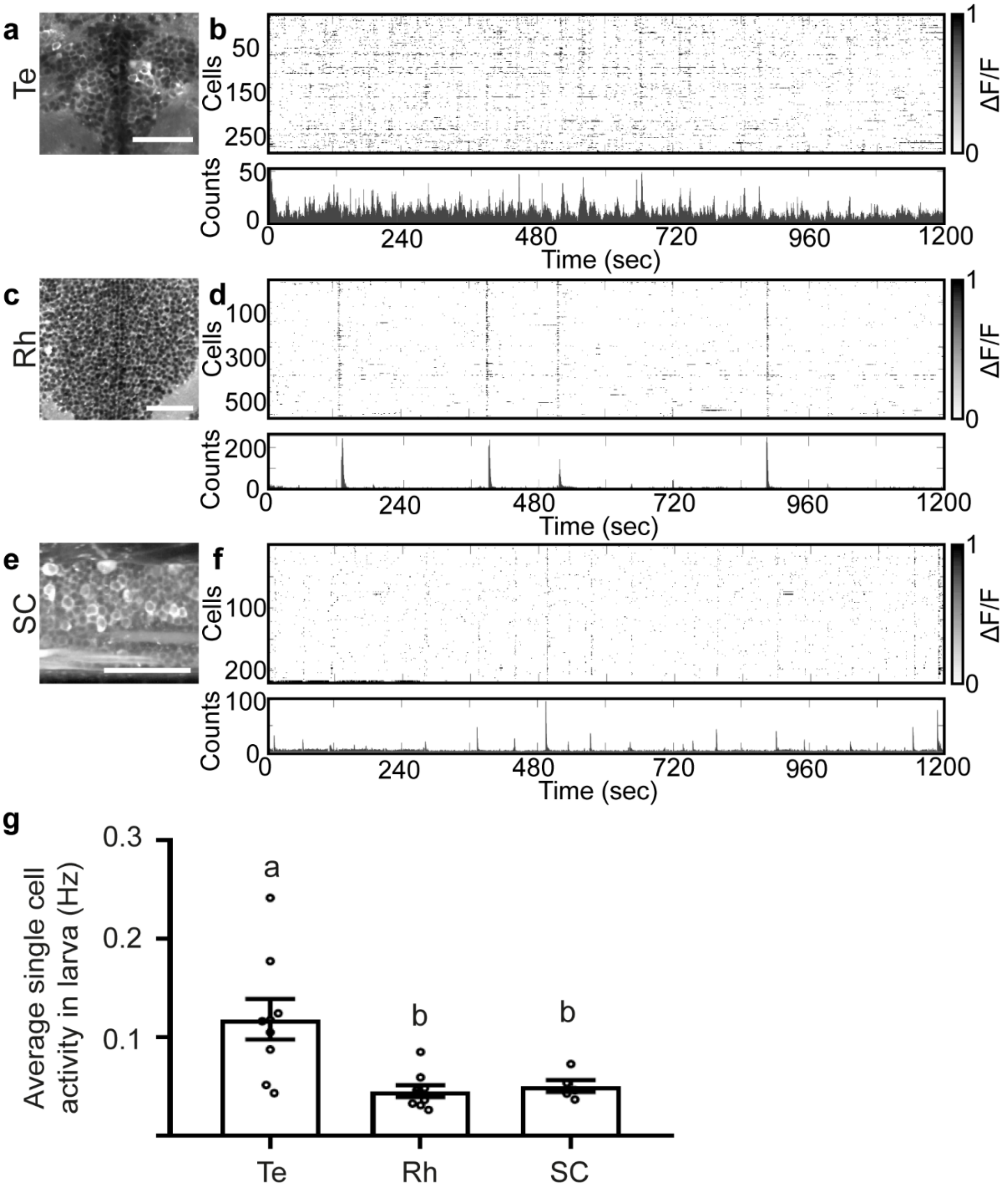

Increased single cell activity in the telencephalon. Representative GCaMP5 expression in neurons of the telencephalon (**a**, Te, n=9 larvae), rhombencephalon (**c**, Rh, n=9 larvae) and spinal cord (**e**, SC, n=5 larvae). Example of a raster plot of ΔF/F in the Te (**b**), Rh (**d**), and SC (**f**). Grayscale: ΔF/F amplitude. Bottom: histogram of the Ca^{2+} transients of all cells. (**g**) Increased spontaneous neuronal activity in the Te neurons. P=0.002, F=8.33,

degrees of freedom=2, determined by one-way ANOVA followed by a Tukey test. Letters indicate significant changes. Values are represented as dot plots and means ± SEM. Scale bar = 50 μm.
