# Supplementary material for: Sleep increases chromosome dynamics to enable reduction of accumulating DNA damage in single neurons
Source: Nat Commun. 2019 Mar 5;10:895. doi: 10.1038/s41467-019-08806-w (PMC6401120; doi:10.1038/s41467-019-08806-w)
Supplement: Supplementary file 2 — Description of Additional Supplementary Files [file 41467_2019_8806_MOESM2_ESM.pdf]

Description of Additional Supplementary Files

File Name: Supplementary Movie 1
Description: Imaging and analysis of chromosome dynamics in the brain of live zebrafish larvae

File Name: Supplementary Movie 2
Description: Imaging of telomere and centromere dynamics in single spinal cord neuron of live zebrafish larvae

File Name: Supplementary Movie 3
Description: Representative imaging of chromosome dynamics in single telencephalic neuron of live zebrafish larvae during the day.

File Name: Supplementary Movie 4
Description: Representative imaging of chromosome dynamics in single telencephalic neuron of live zebrafish larvae during the night.
