# Supplementary material for: Sleep increases chromosome dynamics to enable reduction of accumulating DNA damage in single neurons
Source: Nat Commun. 2019 Mar 5;10:895. doi: 10.1038/s41467-019-08806-w (PMC6401120; doi:10.1038/s41467-019-08806-w)
Supplement: Supplementary file 7 — Reporting Summary [file 41467_2019_8806_MOESM7_ESM.pdf]

nature research

Corresponding author(s): Lior Appelbaum

Reporting Summary

Nature Research wishes to improve the reproducibility of the work that we publish. This form provides structure for consistency and transparency in reporting. For further information on Nature Research policies, see Authors & Referees and the Editorial Policy Checklist.

Statistical parameters

When statistical analyses are reported, confirm that the following items are present in the relevant location (e.g. figure legend, table legend, main text, or Methods section).

n/a	Confirmed	
☐	☒	The exact sample size (*n*) for each experimental group/condition, given as a discrete number and unit of measurement
☐	☒	An indication of whether measurements were taken from distinct samples or whether the same sample was measured repeatedly
☐	☒	The statistical test(s) used AND whether they are one- or two-sided<br>*Only common tests should be described solely by name; describe more complex techniques in the Methods section.*
☐	☒	A description of all covariates tested
☐	☒	A description of any assumptions or corrections, such as tests of normality and adjustment for multiple comparisons
☐	☒	A full description of the statistics including central tendency (e.g. means) or other basic estimates (e.g. regression coefficient) AND variation (e.g. standard deviation) or associated estimates of uncertainty (e.g. confidence intervals)
☐	☒	For null hypothesis testing, the test statistic (e.g. *F*, *t*, *r*) with confidence intervals, effect sizes, degrees of freedom and *P* value noted<br>*Give P values as exact values whenever suitable.*
☒	☐	For Bayesian analysis, information on the choice of priors and Markov chain Monte Carlo settings
☒	☐	For hierarchical and complex designs, identification of the appropriate level for tests and full reporting of outcomes
☐	☒	Estimates of effect sizes (e.g. Cohen's *d*, Pearson's *r*), indicating how they were calculated
☐	☒	Clearly defined error bars<br>*State explicitly what error bars represent (e.g. SD, SE, CI)*

Our web collection on statistics for biologists may be useful.

Software and code

Policy information about availability of computer code

Data collection	Zeiss LSM710 upright confocal/two-photon microscope
Data analysis	Imaris software (Bitplane AG, Zurich, Switzerland), MATLAB software (Mathworks, Natick, Massachusetts, USA), Cell Counter plugin in ImageJ

For manuscripts utilizing custom algorithms or software that are central to the research but not yet described in published literature, software must be made available to editors/reviewers upon request. We strongly encourage code deposition in a community repository (e.g. GitHub). See the Nature Research guidelines for submitting code & software for further information.

Data

Policy information about availability of data

All manuscripts must include a data availability statement. This statement should provide the following information, where applicable:

- Accession codes, unique identifiers, or web links for publicly available datasets
- A list of figures that have associated raw data
- A description of any restrictions on data availability

The data that support the findings of this study are available from the corresponding author upon reasonable request

Field-specific reporting

Please select the best fit for your research. If you are not sure, read the appropriate sections before making your selection.

☒ Life sciences ☐ Behavioural & social sciences ☐ Ecological, evolutionary & environmental sciences

For a reference copy of the document with all sections, see nature.com/authors/policies/ReportingSummary-flat.pdf

Life sciences study design

All studies must disclose on these points even when the disclosure is negative.

Sample size	Initially, experiments were performed three times independently with minimum of three larvae per sample. In most cases, the sample size was larger then three.
Data exclusions	Data was not excluded
Replication	Experiments were performed at least three times independently
Randomization	The larvae and cells were selected randomly.
Blinding	Data analysis conducted blindly by investigator that did not collect the data.

Reporting for specific materials, systems and methods

Materials & experimental systems

n/a	Involved in the study
☒	☐ Unique biological materials
☐	☒ Antibodies
☒	☐ Eukaryotic cell lines
☒	☐ Palaeontology
☐	☒ Animals and other organisms
☒	☐ Human research participants

Methods

n/a	Involved in the study
☒	☐ ChIP-seq
☒	☐ Flow cytometry
☒	☐ MRI-based neuroimaging

Antibodies

Antibodies used	rabbit anti-EGFP (SC-8334, Santa Cruz Biotechnology, Santa Cruz, CA), mouse anti-γH2AX (GTX127342, GeneTex), donkey polyclonal secondary anti-rabbit IgG H&L (Alexa Fluor® 488, ab150061, Abcam), donkey anti-mouse IgG H&L (Alexa Fluor® 594, ab150064, Abcam).
Validation	rabbit anti-EGFP- "recommended for detection of GFP and GFP mutant fusion proteins by WB, IP, IF and ELISA"<br>mouse anti-γH2AX-"Applications: ICC/IF, IHC, IHC-Fr, IHC-P, WB, IHC-Wm. Species reactivity: Zebrafish"<br>anti-rabbit IgG H&L and anti-mouse IgG H&L-"By immunoelectrophoresis and ELISA this antibody reacts specifically with rabbit IgG and with light chains common to other rabbit immunoglobulins. No antibody was detected against non-immunoglobulin serum proteins. Less than 0.1% cross reactivity to bovine, chicken, goat, human, mouse, pig and rat IgG was detected. This antibody may cross react with IgG from other species".

Animals and other organisms

Policy information about studies involving animals; ARRIVE guidelines recommended for reporting animal research

Laboratory animals	Adult wilt type zebrafish (male and female), Transgenic lines: tg(uas:EGFP-Terfa), tg(uas:RCaMP1b), tg(HuC:Gal4), tg(fli:EGFP), tg(mbp:EGFP), tg(fli:Gal4), and tg(HuC:GCaMP5). Mutant line: aanat2-/-
Wild animals	*Provide details on animals observed in or captured in the field; report species, sex and age where possible. Describe how animals were caught and transported and what happened to captive animals after the study (if killed, explain why and describe method; if released, say where and when) OR state that the study did not involve wild animals.*

Field-collected samples

For laboratory work with field-collected samples, describe all relevant parameters such as housing, maintenance, temperature, photoperiod and end-of-experiment protocol OR state that the study did not involve samples collected from the field.
